# Editorial: Democratizing and Innovating Public Health Nutrition Research

**DOI:** 10.3389/fpubh.2022.923777

**Published:** 2022-07-04

**Authors:** Terry T.-K. Huang

**Affiliations:** Center for Systems and Community Design and NYU-CUNY Prevention Research Center, Graduate School of Public Health and Health Policy, City University of New York, New York, NY, United States

**Keywords:** obesity, food insecurity, global health, innovation, health disparities, mental health, diet, non-communicable disease (NCD)

Poor nutrition continues to be one of the major risk factors for morbidity and mortality worldwide. Non-communicable diseases (NCDs), including highly prevalent conditions such as heart disease, stroke, diabetes, respiratory disease and certain cancers, now kill 41 million people each year, representing 71% of all deaths globally (1). A significant factor mediating the effect of poor nutrition on NCDs is obesity. The global prevalence of obesity, a consequence of poor nutrition to a great extent, has tripled since 1975 (1, 2) It is estimated that, in 2016, 39 and 13% of adults aged 18 years and over worldwide were affected by overweight and obesity, respectively. In addition, over 340 million children aged 5–19 years in 2016 and 39 million children aged <5 years in 2020 suffered from either overweight or obesity (3) These numbers are likely to be much higher today, especially given the impact of the COVID-19 pandemic (4).

The COVID-19 pandemic has yet again put obesity in the spotlight. Indeed, obesity is not only a risk factor for NCDs, it is also a risk factor for morbidity and mortality associated with infectious diseases such as COVID-19, including higher risks of severe disease, hospitalization, need for mechanical ventilation and death (5). Obesity may also predispose individuals to increased susceptibility for a range of infections, possibly through impairment of immune responses and vitamin D deficiency (6). However, more research is warranted to understand all the mechanistic pathways via which obesity raises the risk for infectious disease.

Despite the rising prevalence of obesity and NCDs, it is important to recognize that these conditions co-exist with undernutrition in many parts of the world. In low- and middle-income countries, childhood overweight and obesity are rising concurrently with a high prevalence of stunting (28%), wasting (8.8%) and underweight (17.4%) (7). In both developed countries such as the U.S. as well as emerging economies, food insecurity is also real, particularly among minority and under-resourced populations, and often co-occurs with overweight and obesity in the same individuals and/or households (8, 9). While protein-energy deficiency is a critical factor in hunger among populations in the poorest countries (10), the co-occurrence of food insecurity and obesity in the U.S. and emerging economies is often associated with energy-dense but nutrient-poor diets (micronutrient deficiency is present in both cases) (11, 12). The combined challenge of food insecurity, undernutrition and obesity looms even larger today in light of the growing refugee crisis around the world. The UN estimated that, globally, 89.3 million people had been forcibly displaced at the end of 2021 (certainly higher now in 2022 due to the war in Ukraine) (13).

Given the aforementioned challenges in public health nutrition, the need for new research development and research dissemination is greater than ever. I accepted the role of specialty chief editor for the new Public Health and Nutrition section of *Frontiers in Public Health* a year ago because I saw a new opportunity to advance and accelerate the science to address these challenges. Specifically, this new section of the journal has been designed to expand the global footprint of public health nutrition research by shedding light on research coming from different parts of the world, including Africa, Latin America and the Caribbean, South and Southeast Asia, and Eastern Europe and the Middle East. In addition, there is a need to push the boundaries in public health nutrition research. For example, research using new methods, such as systems science, implementation and dissemination science, and novel approaches to community-engaged and community-centered research, or research using new frameworks, such as those coming from political science, ecology, management science, among others, is particularly encouraged. The field would also benefit from more action-oriented research in areas such as policy and technology-enabled solutions. Last but not least, as an open-access journal, there is an opportunity to ensure that null and negative findings of research can be part of our collective knowledge base, as long as the research is rigorously conducted and clearly presented. We must learn from both what works and what does not.

In this inaugural issue of Insights in Public Health and Nutrition, five papers are featured to illustrate either novel areas and/or underserved populations in public health nutrition research. The paper by Sparling et al. makes a strong case for why we must not continue to work in siloes between the field of nutrition and the field of mental health. Indeed, nutrition has an impact on mental health and mental health has an impact on nutrition. The application of a synergistic framework is needed to better advance research linking the two fields and solutions aimed at outcomes in both spheres. The paper by Hagedorn-Hatfield et al. challenges our notion of college-age young adults as healthy and invincible. Indeed, food insecurity is prevalent in this population and many are living independently for the first time. Health behaviors and outcomes acquired during this critical developmental stage may set the path for many regarding future adult health. Furthermore, the lack of fiber intake is a common characteristic of poor nutrition. Harris et al. makes a provocative case for increasing the supply and intake of high-amylose wheat flour, a source of resistant starch fiber, to improve diet-related health.

Finally, two additional papers raise important issues related to the implementation of nutrition-related health interventions. The first paper, by Watanabe and Seale, focuses our attention on a much understudied and underserved population in the U.S.—Native Hawaiians and Pacific Islanders—despite the well-documented higher prevalence of NCDs in this group. The paper discusses potential reasons for the low adherence to statin therapy in this group, including possibly higher muscle toxicity, and the potential of culturally aligned behavioral interventions, such as Hula dance (involving controlled rhythmic movements), as alternative or adjuvant treatment while lowering the dose intensity of statins. The second paper, by Kerins et al., showcases a mixed methods study on organizational factors that may be critical to the implementation of a calorie-posting policy in public hospitals in Ireland. The study demonstrates that adoption of a public health policy is not enough. Indeed, more attention is needed on multi-component and multi-level strategies to improve policy implementation fidelity and impact.

In brief, as we approach the one-year anniversary of the Public Health and Nutrition section of *Frontiers in Public Health*, we are energized by the positive response received thus far from researchers around the world. While there is much work to be done to improve nutrition and health at the population level, advances in science and technology are happening more rapidly than ever. Our section of the journal is here to capture and amplify these dynamic and innovative developments. In turn, we hope to make a lasting impact on enhancing population health and reducing health disparities.

## Author Contributions

The author confirms being the sole contributor of this work and has approved it for publication.

## Funding

The author is supported in part by grants from the National Cancer Institute (R01CA206877) and the Centers for Disease Control and Prevention (U48DP006396).

## Conflict of Interest

The author declares that the research was conducted in the absence of any commercial or financial relationships that could be construed as a potential conflict of interest.

## Publisher's Note

All claims expressed in this article are solely those of the authors and do not necessarily represent those of their affiliated organizations, or those of the publisher, the editors and the reviewers. Any product that may be evaluated in this article, or claim that may be made by its manufacturer, is not guaranteed or endorsed by the publisher.
